# D1018 with higher stability and excellent lipopolysaccharide binding affinity has potent anti-bacterial and anti-inflammatory activity

**DOI:** 10.3389/fmicb.2022.1010017

**Published:** 2022-12-01

**Authors:** Runzhe Wu, Xunxi Dong, Qiang Wang, Zirui Zhang, Jianhua Wang, Xiao Wang

**Affiliations:** ^1^School of Medicine, Ningbo University, Ningbo, Zhejiang, China; ^2^School of Food and Pharmacy, Ningbo University, Ningbo, Zhejiang, China; ^3^The Affiliated Hospital of Medical School, Ningbo University, Ningbo, Zhejiang, China

**Keywords:** antimicrobial peptide D1018, antibacterial, stability, LPS binding, *Escherichia coli*

## Abstract

*Escherichia coli* (*E. coli*) infection and LPS-induced inflammation are still of severe threat to human health. With the increasing problem of antibiotic resistance, there is a desperate need to develop new approaches to solve the problem. Antimicrobial peptide (AMP) IDR-1018 exhibited potential antimicrobial and immunoregulation activity. However, moderate antimicrobial efficiency and susceptibility to protease cleavage limited its therapeutic application. Therefore, the derived 1018M which has better activity against MRSA and whole sequence D-amino acids substitution peptides (D1018 and D1018M) were synthesized in this study. The resistance of D1018 and D1018M against tested proteases increased (2–4 times), particularly in D1018. The antibacterial activity of D1018 was the same as that of the parent peptide IDR-1018, but the antimicrobial activity of D1018M was slightly increased (2-fold). Though the hemolysis of IDR-1018 and D1018 was about 2%, at the concentration of 8×MIC, the cytotoxicity of IDR-1018, D1018, and 1018M was negligible. The peptides could interact with *E. coli* cell wall and cytoplasmic membrane, penetrate the membrane, cause leakage of contents, and disrupt genomic DNA. Among them, D1018 is the most prominent one. In addition, IDR-1018 and D1018 showed potent binding ability to LPS, thus leading to excellent inhibition capacity to LPS-induced proinflammation response. Taken together, these data demonstrate that D1018 is a promising peptide candidate for the treatment of *E. coli* infection.

## Introduction

*Escherichia coli* (*E. coli*) is a natural bacterial parasite in the intestinal tracts (Ishii and Sadowsky, 2016). However, as a major cause of enteritis, urinary tract infection, neonatal meningitis, bacteriaemia, and septicemia (Allocati et al., 2013), it is still associated with high mortality (Sandra et al., 2021). Attributed to long-term pressure of overuse and abuse of antibiotics, varying degrees of resistance have been developed in *E. coli* strain. In China, the national average resistance rate of *E. coli* to quinolones in 2020 was 50.7% while the one to third-generation cephalosporins was 51.6% (CRASS, 2022). Consequently, to find out novel antimicrobial candidates is an urgent need.

Though antibiotic-treated patients could have had a higher survival rate, they had still showed higher lipopolysaccharide (LPS) levels than those antibiotic-free ones. LPS, composed of polysaccharides and lipids, is a major component of the gram-negative bacteria membrane. As the bacteria die, plasma LPS increases despite decreasing bacteremia, which may be lethal for sensitized patients (Holzheimer, 2001). LPS has the capacity of binding to Toll-like receptors (TLRs) of the cell as well as activating various downstream signaling pathways, leading to the synthesis of interleukin (IL)-1β, tumor necrosis factor alpha (TNF-α), IL-6, and other proinflammatory cytokines (Wells et al., 2009; Chunhua et al., 2022) Not only can they arouse an inflammatory response and launch fever in infected bodies, but also promote the adhesion of endothelial cells and white blood cells *via* regulating the expression of adhesion molecules, thus leading to collateral damage to tissues and even septicemia. Hence, the novel candidates with both antibacterial and LPS binding activities, which may inhibit the infection and proinflammatory response at the same time, will be of great contribution to preventing and curing diseases.

As essential components of innate immunity, antimicrobial peptides (AMPs) contribute to the first line of defense against infections. Among them, innate defense regulators (IDRs) 1018 (IDR-1018, VRLIVAVRIWRR-NH2), a synthetic derivative of natural bovine host defense peptides bactenecin, displays multiple biological activities including antibacterial/biofilm and immunoregulation functions (Haney Evan et al., 2018). These properties indicate that IDR-1018 might possess the potential of being a beneficial candidate for the treatment of hyperinflammatory for *E. coli* infections. However, its antibacterial activity (MIC = 5–260 μg/mL) remains to be promoted compared to the traditional antibiotics (de la Fuente-Núñez et al., 2014). Moreover, the activity of IDR-1018 could be decreased 2–16 times when exposed to inappropriate temperature, pH, or protease environment, which seriously affected the subsequent application (Zhou et al., 2021).

Therefore, in this study, the derived peptide 1018 M, which is more stable and potent against MRSA and its biofilm, was evaluated for its anti-*E. coli* and LPS binding ability. Additionally, since the target of protease *in vivo* was L-amino acids instead of D-amino acids, the latter were employed as an alternative to make D1018 and D1018M based on IDR-1018 and 1018M. Subsequently, the binding affinity, antibacterial activity, stability, biological characteristics and mechanism of antimicrobial activity of 1018M, D1018, and D1018M were explored for the first time.

## Materials and methods

### Bacterial strains and cell lines

*Escherichia coli* ATCC25922 were purchased from the American Type Culture Collection (ATCC) (VA, United States). The RAW 264.7 and Vero cells were donated by Dr. Guo Hua and Dr. Zhang Jili (Ningbo University).

### The preparation of peptides

Retaining the key binding amino acids, increasing the positive charge and membrane anchor amino acids appropriately, 1018M, taking IDR-1018 as the parent, was designed by substituting amino acid residues (the amino acid residues I4, V5, A6, V7, and I9 were replaced by R, W, W, R, and R), which was reported in our previous research (Zhou et al., 2021). Aiming at improving the stability of IDR-1018 and 1018M, D form amino acids were used as alternatives. IDR-1018 1018M D1018 and D1018M were synthesized by solid-phase synthesis.

### Antimicrobial activity

#### Minimum inhibitory concentration

The MIC values of peptides against *E. coli* ATCC25922 were determined by the microtiter broth dilution method. The bacteria were grown to the mid-logarithmic phase and diluted into the Luria-Bertani (LB) broth at 1 × 10^5^ CFU/mL. Two-fold serial dilutions of peptides were prepared in sterile aqua distillate with a gradient concentration of 640, 320, 160, 80, 40, 20, 10, 5, 2.5, 1.25, and 0.625 μg/mL. A total of 10 μL peptides and 90 μL bacterial suspensions had been added into 96-well plates before the plate was incubated at 37°C for 16–24 h. Polymyxin and the sterile aqua distillate were tested as control. All assays were performed in triplicate. The MIC lied on the lowest peptide concentration at which there is no growth of bacteria observable (Xinyi et al., 2021).

#### Time-kill curves

In order to study the effects of peptides on growth curves of *E. coli* ATCC25922, the mid-log phase bacteria were diluted to 1 × 10^5^ CFU/mL with fresh mediums. Subsequently, 5 mL of bacteria solution and different concentrations of peptides (1×, 2×, 4× MIC) were added to a 50 mL shaking flask. Eventually, the mixtures were cultured at 37°C and 250 rpm. A total of 150 μL samples were taken from each flask at 0, 0.5, 1, 2, 4, 6, 8, 10, 12, and 24 h. The number of bacteria was measured by plate colony count. Whilst, polymyxin at 2 × MIC and peptide solvent without peptide itself were taken as positive and negative control. All tests were run in triplicate (Na et al., 2019).

### Stability, toxicity and resistance

#### Protease stability

In order to acknowledge protease stability of the peptides, they were in 4 h incubation with pepsin (3,000 U/mg, pH 2.0) and trypsin (250 U/mg, pH 8.0) (10:1, w/w, the concentration of peptide and protease were 320 μg/mL and 32 μg/mL) solutions, respectively, at 37°C. The untreated peptides and buffer solution were used as control. The antibacterial activity of treated peptides against *E. coli* ATCC25922 was determined by MIC determining assay (Zhou et al., 2021). All assays were carried out in triplicate.

#### Hemolysis

Hemolytic activity was estimated by determining hemoglobin released from healthy mouse red blood cells. Blood cells were washed three times in sterile PBS (10 mM, pH 7.4) and centrifuged at 1500 rpm for 5 min at 4°C. Then 100 μL red blood suspensions (8%, v/v) and 100 μL peptides at different concentrations (0.25–128 μg/mL) were mixed. Afterward, the mixtures were incubated at 37°C for 1 h and centrifuged at 1500 rpm for 5 min. Ultimately, the absorbance of supernatants was measured at 540 nm. PBS and 0.1% Triton X-100 were used as the negative and the positive control, respectively. Hemolysis (%) = [(OD540 nm of the treated sample-OD540 nm of the negative control)/ (OD540 nm of positive control-OD540 nm of negative control)] × 100%. Three replicates were carried out for each condition (Na et al., 2019).

#### Cytotoxicity

The performance of the Cell Counting Kit-8 (CCK-8) assay was applied to investigate the effect of peptides on the viability of murine RAW264.7 macrophage cells and Vero cells in accordance with the previous method (Zhou et al., 2021). Cells (2.5 × 10^4^ cells/well) were added into 96-well plates and incubated at 37°C in a humidified 5% CO_2_ environment for 24 h. Then various concentrations (0.15–20 μg/mL) of peptides were mixed with cells. Sterile aqua distillate was used as control. Eventually, 10 μL WST-8 solutions were added into each well. After 4 h of incubation at 37°C, a microplate reader was applied to measure the absorbance of each sample at 460 nm. The following formula was used to calculate cell viability: Cell viability (%) = OD 460 nm of treated sample/OD 460 nm of control × 100%.

#### Resistance

The development of resistance against *E. coli* ATCC25922 of these peptides was performed by MIC assay. Mid-log phase of bacteria (1 × 10^5^ CFU/mL) (90 μL/well) was added to 10 μL peptides solution (128, 64, 32, 16, 8, 4, 2, 1, 0.5, 0.25 μg/mL). Polymyxin were used as control. After incubation at 37°C for 16–18 h, bacteria form 1/4 MIC well were used to inoculate in fresh culture. The MIC values were continually determined as described above. The serial passaging was repeated for 11 days.

### Effects of peptides on cell wall and membrane

#### Scanning/transmission electron microscope observations

Mid-log phase *E. coli* ATCC25922 (1 × 10^8^ CFU/mL) treated with 4× MIC of peptides for 2 h at 37°C. After washed by PBS twice, 2.5% glutaraldehyde was used to fix bacteria at 4°C for 12 h. For SEM, the bacteria were dehydrated with a series of concentrations of ethanol solutions (20, 50, 70, 85, 95, and 100%) and dried by CO_2_ overnight then. Samples were observed with S4800 SEM followed by sputtering gold–palladium. As for TEM, the bacteria were further fixed with 1% OsO_4_ for 1 h. Then samples were dehydrated with a graded acetone series (50, 70, 85, 95, and 100%) for 7 min each time. Subsequently, the bacteria were immersed in epoxy resin, embedded in capsules, polymerized at 45°C and 65°C for 3 h and 24 h, respectively. These sections were acquired by ultramicrotome, followed by staining with 1% uranyl acetate. Images were captured by Hitachi H-7650 TEM (Na et al., 2019).

#### Membrane permeabilization analysis

To assess bacterial cell membrane permeabilization activity of peptides, the propidium iodide (PI) uptake assay was carried out. Mid-log phase *E. coli* ATCC25922 (1 × 10^8^ CFU/mL) were incubated with 1 × MIC, 2 × MIC, and 4 × MIC peptide solutions for 5, 30, and 120 min at 37°C, respectively. The bacteria without treatment were used as negative controls. Samples were washed twice before they were incubated in 50 μg/mL PI for 15 min. Eventually, the fluorescence was analyzed by FACS Calibur Flow Cytometer (BD, United States; Na et al., 2017).

### Effects of peptides on bacterial genomic DNA

#### Gel retardation assay

Gel migration experiment was carried out to determine the interaction of peptides and *E. coli* genomic DNA. Genomic DNA was obtained from the bacterial genome extraction kit. A series of gradient concentrations of peptides were mixed with DNA, respectively. The mixture was incubated at room temperature for 10 min and analyzed by electrophoresis on a 0.8% agarose gel afterward.

#### Circular dichroism spectroscopy

Furthermore, the secondary structure changes of *E. coli* ATCC25922 genomic DNA after mixed with peptides were analyzed by CD spectra. The peptides (40 μg/mL) and genomic DNA (150 μg/mL) were incubated for 10 min at room temperature before the samples were loaded into a cuvette with 1.0-mm path length. Finally, the samples were tested on a CD spectrometer (J-1700 CD). The spectra were recorded from 220 to 320 nm at 25°C with a 10 nm/min scanning speed (Ning and Donghui, 2018).

#### Lipopolysaccharide binding affinity

The binding affinity of peptides to the lipid A region of LPS was determined by the Tachypleus Amebocyte Lysate (TAL) method. A gradient concentration of peptides was volume-equally incubated with 0.4 ng/mL control standard endotoxin (CSE) resolved in TAL reagent water in test tubes at 37°C for 30 min. Afterward, they were added into a 96-well plate and reacted with End-Point Chromogenic Endotoxin Test Kit (With Diazo Coupling). The absorbance was tested at 545 nm with a microplate reader. A gradient concentration of control standard endotoxin was used to make the standard curve. All tests were run in triplicate (Pearson, 1979).

#### Bioassay for cytokines

Enzyme-linked immunosorbent assay (ELISA) was carried out to quantitate the concentration of cytokines accumulated in RAW264.7 cells’ cultural supernatant. Briefly, 1 μg/mL LPS and different concentrations of peptides were mixed and incubated at 37°C for 30 min. RAW264.7 cells were added into 12-well plates (1.875 × 10^5^ cells/well). After cultured for 24 h, the cells were incubated with the mixture of LPS and peptides at 37°C in a humidified 5% CO_2_ environment for another 24 h. Supernatants harvested from different treatments were obtained and tested by different ELISA kits (mouse IL-6, TNF-α; Mire-Sluis and Thorpe, 1998). The 96 well plates were read on a microplate reader at a wavelength of 450 nm, 570 nm, and 630 nm. LPS-free cells were used as standard control. All tests were run in triplicate.

## Results

### Antimicrobial activity

#### MIC

As exhibited in Table 1, MIC of IDR-1018, 1018M and D1018 against *E. coli* ATCC25922 was 8 μg/ mL while that of D1018M was 4 μg/ mL. D1018M owned stronger potential antibacterial activity against *E. coli* ATCC25922. However, the antimicrobial activity of peptides was not so good as that of polymyxin (MIC = 1.56 μg/mL).

**Table 1:** The MICs of IDR-1018, D1018, 1018M, D1018M, and polymyxin against *E. coli* ATCC25922.

Drugs	MIC (μg/mL)
	ATCC25922
IDR-1018	8
D1018	8
1018 M	8
D1018M	4
Polymyxin	1.56

### Time-killing curves

**Figure 1:**
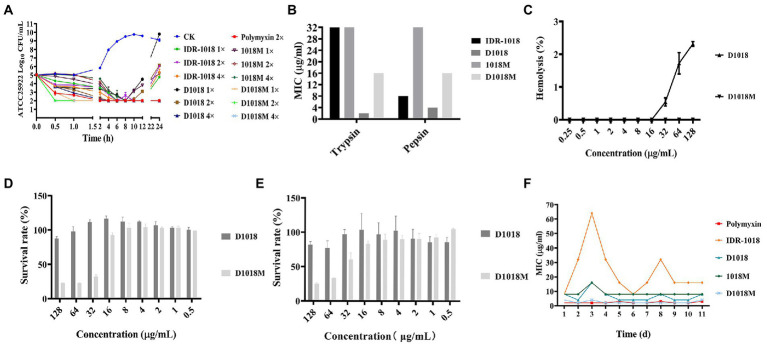
The killing curves, stability, toxicity and resistance of peptides. **(A)** The killing curves of IDR-1018, D1018, 1018M, and D1018M. **(B)** The protease stability of IDR-1018, D1018, 1018M, and D1018M. **(C)** The hemolysis of D1018 and D1018M. **(D)** The cytotoxicity of D1018 and D1018M against RAW264.7 cells. **(E)** The cytotoxicity of D1018 and D1018M against Vero cells. **(F)** The resistant of *E. coli* ATCC25922 against IDR-1018, D1018, 1018M, and D1018M.

The time dose dependence of peptides against *E. coli* ATCC25922 was studied in this research. The results shown in Figure 1A demonstrated that after treatment with 1×, 2×, 4 × MIC of D1018M (no bacterial growth) and 2×, 4 × MIC of D1018 (1.72, 2.11 log CFU reduction, respectively), the amount of ATCC25922 dropped dramatically within 1 h, which was superior to the parent peptide (0.99, 1.18 and 1.39 log CFU reduction at 1×, 2×, 4 × MIC, respectively). Moreover, antimicrobial efficiency of 1×, 2×, and 4 × MIC of D1018M were all superior to 2 × MIC of polymyxin as well. The antibacterial activity of all IDR-1018 and its derived peptides could last for 8–12 h with high efficiency. Compared with the parental peptide and traditional antibiotic polymyxin, we found that the novel peptide D1018M has the advantages of rapid and longer antibacterial effects.

### Stability, toxicity and resistance

#### Protease stability

As is exhibited in Figure 1B, the protease stability of IDR-1018 and its derivative peptides was tested. After exposed to pepsin and trypsin for 4 h, the antibacterial activity of D1018 remained unchanged or even lower (MIC = 4 and 2 μg/mL), while the anti-*E. coli* ability of IDR-1018, 1018M, and D1018M declined 2–4, 4, and 4 times. In general, D1018 showed the best tolerance to protease among all peptides.

#### Hemolysis and cytotoxicity

The toxicity of peptides to mouse eukaryotic cells, macrophagocyte RAW 264.7 and Vero cells was determined. The hemolysis rates of IDR-1018, which had been reported in our previous research, were 0.386, 2.136, and 2.651% at the concentrations of 32, 64, and 128 μg/mL (Zhou et al., 2021) while those of D1018 were 0.542, 1.724, 2.316% at the same concentrations (Figure 1C). Nevertheless, hemolysis of 1018M (Zhou et al., 2021) and D1018M was 0% at all tested concentrations (Figure 1C), which was apparently lower than parental peptides. All the results indicated that D amino acid substitution had little effect on peptide hemolysis. The cytotoxicity results of peptides were shown in Figures 1D,E, the cell survival rate in D1018M group was 22.9, 22.8, 32.6, 92.5 and 25.3%, 33.3, 60.7, 83.0% at the concentrations of 128, 64, 32, and 16 μg/mL, while the survival rates were 95.9%, 81.7–87.6, and 97.3% after treatment with 128 μg/mL IDR-1018 (Zhou et al., 2021), D1018 (Figures 1D,E), and 1018M (Zhou et al., 2021), respectively. The results indicated that peptides IDR-1018, 1018M and 1018M basically had no cytotoxicity against murine macrophage cells, when the concentration was up to 16 times of MIC. However, for D1018M, the safe concentration of murine macrophage cells was only 4 times of MIC.

#### Resistance

After 11 serial passages of *E. coli* ATCC25922 in the presence of these peptides, the MIC values did not increase over time except some test errors. IDR-1018 group was even more pronounced, which might be due to the instability of this peptide. There was also no change in MIC for polymyxin (Figure 1F). The results indicated that all these peptides had no resistance, which might serve as good novel antimicrobial agents against *E. coli.*

### Effects of peptides on cell wall and membrane

#### Scanning/transmission electron microscope observations

**Figure 2:**
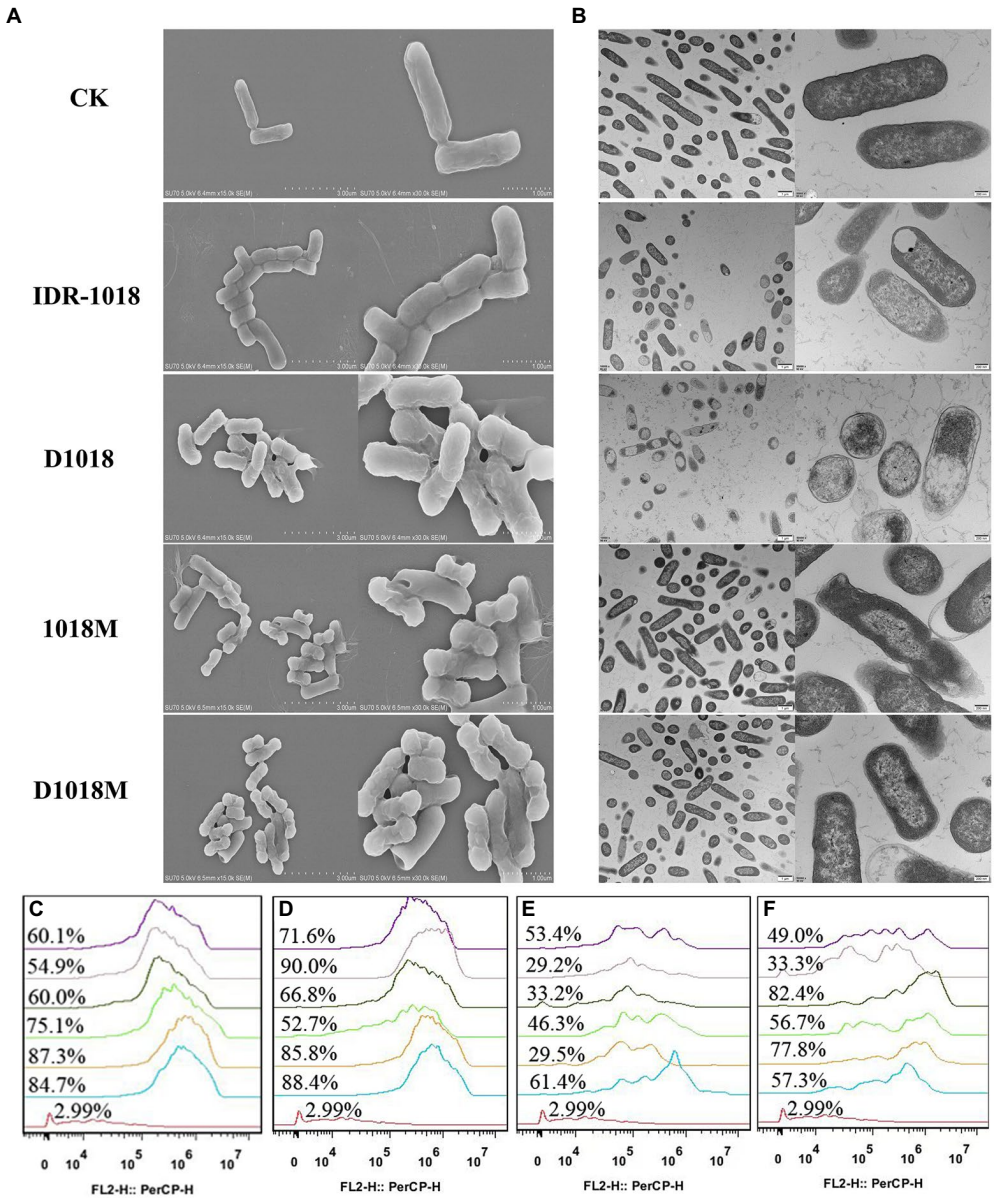
Effects of peptides on cell wall and membrane. **(A)** Scanning electron microscope. **(B)** Transmission electron microscope. Flow cytometric analysis of PI-staining in *E. coli* ATCC25922 cells treated with 1×, 2×, or 4× MIC IDR-1018 **(C)**, D1018 **(D)**, 1018M **(E)**, and D1018M **(F)** for 30 or 120 min, respectively. Red line: no peptide, negative control; Blue line: treatment with 1 × MIC peptides for 30 min; Orange line: treatment with 1 × MIC peptides for 120 min; Green line: treatment with 2 × MIC peptides for 30 min; Dark green line: treatment with 2 × MIC peptides for 120 min; Pink line: treatment with 4 × MIC peptides for 30 min; Purple line: treatment with 4 × MIC peptides for 120 min.

The changes in bacteria morphology, integrity and cellular structure after treatment with peptides were observed by SEM and TEM. Compared to the control group, the cell wall of *E. coli* ATCC25922 treated with peptides was resolved gradually. Cell edges were blurred and lots of holes appeared on the surface of the bacteria. Shrunken and bubbling bulges of bacteria cells were observed. These phenomena were more obvious in the groups of D1018, 1018M, and D1018M, which revealed that they were more destructive than parental peptide IDR-1018 (Figure 2A). Additionally, *E. coli* internal ultrastructure image was captured by TEM (Figure 2B). Most of the bacterial morphology was normal and intact in the untreated control group. After exposure to the peptides, severe damage to bacterial cell walls, membranes, and cytoplasm was observed, especially to the cytoplasm. Light-colored areas or even cavities formed in cytoplasm, which meant cellular contents leakage of treated bacteria. Almost 100, 50, and 40% of bacteria were destroyed by the treatment of D1018, IDR-1018, and D1018M which indicated better performance of these peptides.

#### Membrane permeabilization analysis

Prodium iodide (PI), as a sort of nucleic acid fluorescent, dye can penetrate the damaged bacterial cell membrane and then lable the bacteria. After treatment with peptides and PI, the bacteria were determined by flow cytometry. As exhibited in Figures 2C–F, the untreated bacterial cell membrane was intact and impervious with a fluorescence rate of 2.99%. In incubation with 1×, 2×, and 4 × MIC of IDR-1018 for 0.5 h and 2 h, the percentages of PI-permeable bacteria were 54.9–84.3%. The proportions of D1018 were 52.7–90%, better than IDR-1018. While, for 1018M and D1018M, the proportions of fluorescence-positive bacteria were 29.2–61.4% and 33.3–77.8%, respectively. These data illustrated that D1018 had the strongest penetrating ability among all the peptides.

### Effects of peptides on bacterial genomic DNA

#### Gel retardation assay

**Figure 3:**
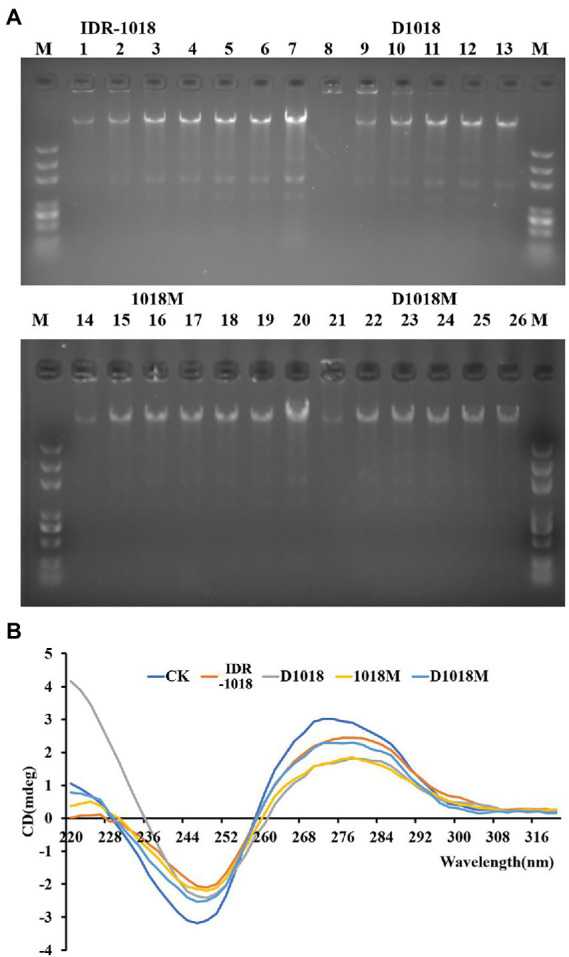
Interaction of peptides with *E. coli* ATCC25922 bacterial genomic DNA. **(A)** Interaction of IDR-1018, D1018, 1018M, and D1018M with bacterial genomic DNA by a gel migration assay. M: DNA marker; 1–6: The concentration of IDR-1018 were 0, 0.625, 1.25, 2.5, 5, and 10 μg/mL, respectively. 8–13: The concentration of D1018 were 0, 0.625, 1.25, 2.5, 5, and 10 μg/mL, respectively. 14–19: The concentration of 1018M were 0, 0.625, 1.25, 2.5, 5, and 10 μg/mL, respectively. 21–26: The concentration of D1018M were 0, 0.625, 1.25, 2.5, 5, and 10 μg/mL, respectively. 7 and 21: Genomic DNA from *E. coli* ATCC25922 with no peptide. **(B)** CD spectra of genomic DNA from *E. coli* ATCC25922 in the presence of IDR-1018, 1018M, D1018, and D1018M. The concentration of peptide and DNA were 40 and 150 μg/mL, respectively.

DNA-interaction properties of peptides and *E. coli* genomic DNA were examined by gel retardation assay. All the peptides could significantly inhibit the migration of *E. coli* ATCC 25922 genomic DNA at the highest tested concentration (10 μg/mL). However, the inhibition ability was not obvious at lower peptide concentrations (Figure 3A). D1018 stood out among all these peptides, showing particularly excellent capability of inhibiting the migration of *E. coli* ATCC 25922 genomic DNA.

### CD spectroscopy

The influence of peptides on *E. coli* genomic DNA secondary structure was further revealed by CD spectrometer, which could determine the morphology changes of DNA after interacted with peptides. The positive and the negative peaks of *E. coli* ATCC25922 normal genomic DNA were at 270 nm and 246 nm, respectively in the CD spectrum. After treatment with peptides, all the positive and negative peaks decreased, especially on D1018 and 1018M. Additionally, D1018 narrowed the negative peak sharply. The changes suggested that the peptides damaged DNA helical structure and weakened base stacking force. The destructive potential of D1018 against DNA conformation was higher than other peptides (Figure 3B).

### Lipopolysaccharide binding affinity

**Figure 4:**
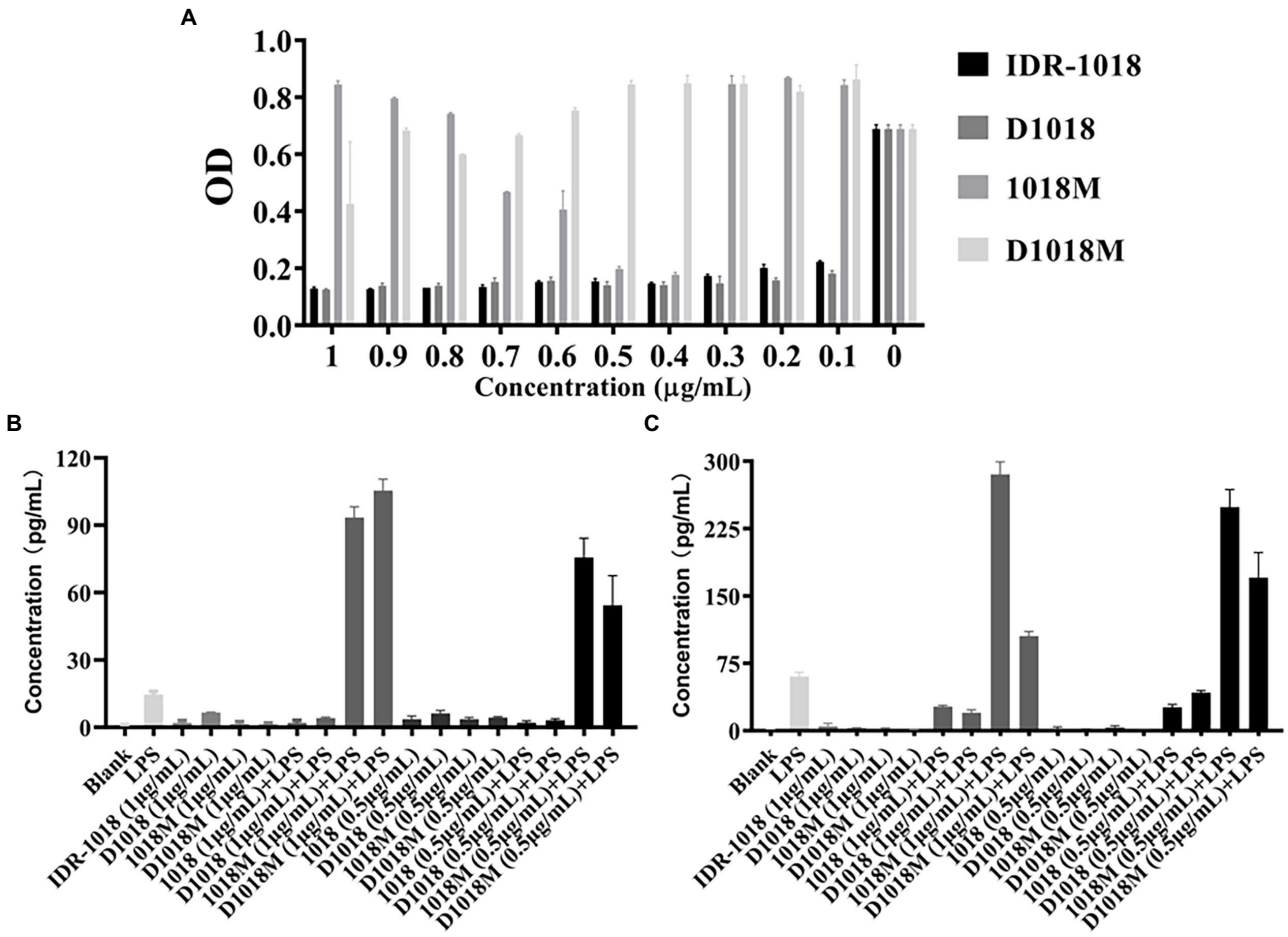
LPS binding affinity of peptides and the effects of binding to LPS-induced proinflammatory response. **(A)** LPS Binding affinity of IDR-1018, 1018M, D1018, and D1018M. Effect of IDR-1018, 1018M, D1018, and D1018M on IL-6 **(B)** and TNF-α **(C)** produced by RAW 264.7 cells.

Tachypleus Amebocyte Lysate (TAL) method was carried out to test the binding affinity of peptides with LPS, the result was shown in Figure 4A. IDR-1018 and D1018 have strong binding affinity with LPS at the lowest tested concentration (0.1 μg/ mL), the binding rates were up to 68 and 74%. While, the best LPS binding concentrations for 1018M was 0.4 μg/ mL (74%) and 0.5 μg/ mL (82%). Moreover, with the decrease and increase of 1018M concentration, the binding ability declined. Among these peptides, D1018M has the weakest binding ability to LPS, with only a 38% binding rate at 1 μg/ mL.

### Bioassay for cytokines

To explore the effects of the peptides on proinflammation cytokines, RAW 264.7 macrophages were challenged with LPS or peptides treated with LPS. As exhibited in Figures 4B,C, the cytokine levels of IL-6 and TNF-α in the LPS control groups were significantly enhanced (60.25 and 14.68 pg/mL, respectively). While, the secretion of cytokines was not promoted in response to all the peptides incubation, which indicated that these peptides are unlikely to trigger inflammation. Moreover, both IDR-1018 and D1018 significantly suppressed the production of TNF-α (26.83–26.11 and 20.09–42.44 pg/mL) and IL-6 (2.02–2.10 and 3.15–4.18 pg/mL). However, after treatment with 1018M and D1018M, TNF-α (248.83–285.45 and 105.49–170.44 pg/mL) and IL-6 (75.63–93.40 and 54.35–105.49 pg/mL) levels increased sharply, even higher than LPS group. This result revealed that IDR-1018 and D1018 could bind to LPS, thus leading to an inhibition in the LPS-induced inflammatory response. Nevertheless, with extremely low combination ability to LPS, 1018M and D1018M not only could not suppress the production of proinflammation cytokines but also oppositely promoted the process.

## Discussion

*E. coli* is still one of the most momentous pathogenic bacteria threatening human health. LPS, the primary toxic component of the outer cell wall of *E. coli*, can be released rapidly and spontaneously during both growth and death of the bacteria, which is still of severe threat to the clinical treatment for infection. Antibiotics have been playing an irreplaceable important role in protecting human beings from pathogens infections for a long time. However, with the extensive use of antibiotics, the emergence of antibiotic resistant bacteria increased dramatically. Therefore, AMPs attracted much attention from researchers for their multi-target and multi-function action mode (Fengjing et al., 2017). IDR-1018, with moderate MIC, minimal hemolytic activity, and significant immunoregulatory activity, was a promising candidate (Michal et al., 2010). Nevertheless, the susceptibility to protease was still one critically defective issue that limited its future success in clinic (Zhou et al., 2021).

It has been demonstrated that the stability of peptides against the degradation of protease could be improved by all or part of D-amino acid substitution, because the target of protease *in vivo* was L-amino acids instead of D-amino acids, trypsin and the plasma proteases possessed much weaker hydrolytic activity toward D-Lys, D-Arg than L-Lys and L-Arg (Lu Jianguang et al., 2018). However, the antimicrobial activity, cytotoxicity, hemolysis, active action mode, anti-inflammatory activity and other properties may be changed by the transformation (Binu et al., 2016; Fengjing et al., 2017). Therefore, in this study, two D-amino acid substitution peptides were designed and synthesized based on IDR-1018 and its derivative peptide 1018M, which had manifested preferable properties in our previous anti-MRSA and its biofilm investigation. Then their antibacterial activity, stability, antibacterial mechanism, LPS binding affinity and immunoregulatory capacity were first studied.

First of all, the antibacterial activity against *E. coli* ATCC25923 was evaluated. MIC of D1018 was not changed compared to its parental peptide after substitution. Moreover, D1018M not only got the lowest MIC (Table 1) but also the best antimicrobial efficiency (Figure 1A) against *E. coli* ATCC25923 among all the tested peptides. Whether D-type amino acid substitution can improve the antimicrobial activity of antimicrobial peptides has always been controversial. Ascaphin-8 maintained its original antibacterial activity when the Lys in the position of 4 and 8 was substituted by D-Lys. However, the same change of Lys10 Lys14 and Lys18 increased Ascaphin-8’s MIC sharply, especially against *Staphylococcus aureus* (*S. aureus*) (Tibor et al., 2005). Intriguingly, the activity of D-Melittin turned out to be slightly lower than Melittin against *E. coli*, while the opposite result was discovered against *S. aureus* (Wade et al., 1990). Therefore, the impact of D amino acid on antibacterial effect is far from absolute, which may be influenced by the change of secondary structure (Shikha and Vipasha, 2020), dynamics of self-assembled (Zhou et al., 2018), or other unknown factors. Further investigation was required in this field.

Barriers to make these peptides become effective drug candidates include some limiting biological properties, such as stability and cytotoxicity. Optimizing and understanding these properties are major approaches to taking full advantage of these peptides. As anticipated, protease cleavage stability of D1018 and D1018M was enhanced significantly against trypsin and pepsin, especially D1018 (Figure 1B), providing the same conclusion as previous researches (Lu Jianguang et al., 2018; Li et al., 2019). In consistency with the study of Li et al., the substitution of D amino acid had no significant effect on peptide hemolysis (Figure 1C). (Li et al., 2019). However, cytotoxicity limited the potential of D1018M (Figure 1D). One possible exploration is that the abundance of D-type aromatic amino acids in D1018M exerts more efficient anchoring ability in the hydrophobic core of the cell membrane (Munhoz Victor et al., 2021). What’s more, the inflammatory response of RAW264.7 cell induced by D1018M also had a profound impact on cell activity. Given the biological property differences between these four peptides, it appears that 1018M was the safest one when administered intravenously. For IDR-1018, D1018 and D1018M, the dosage should be taken into consideration in the subsequent research and application. While oral administration can be tried for D1018 and D1018M because of their resistance against trypsin and pepsin, which will absolutely widen the application area and pave the way for further exploration.

Our previous study has demonstrated that IDR-1018 and D1018M can interact with MRSA cell wall and cytoplasmic membrane, penetrate the membrane, cause leakage of contents, disrupt genomic DNA and then kill the bacteria (Zhou et al., 2021). As such, the anti-*E. coli* mechanism of peptides was elucidated in this study. The shrunken cell wall, damaged cell membrane, permeated PI and blocked genomic DNA indicated that these peptides basically share the same bactericidal mechanism against *E. coli* with MRSA. However, the damage to bacteria cell wall and membrane caused by IDR-1018 and D1018 is more severe which is exhibited in TEM and flow cytometry figures (Figure 2). D-amino acid substitution improves the ability of IDR-1018 to destroy the cell wall and membrane, which is consistent with the result that diastereomers bind 50-100-folds better to negatively-charged PE/PG membranes (Li et al., 2016). However, the phenomenon is not suitable for 1018M, and the previous studies about D-W3R6 and D-CP got similar conclusion as well (Li et al., 2019; Fengjing et al., 2017). Therefore, further studies are needed to elucidate the underlying mechanisms. This is different from the phenomenon of our previous report on MRSA, which may be related to the higher binding affinity to LPS of IDR-1018 and D1018, thus leading to a better interaction with bacterial outer and inner membranes of *E. coli* compared to 1018M and D1018M.

LPS is the major component of gram-negative bacteria’s outer membrane, which could stimulate a proinflammatory cascade and trigger an inflammatory response *via* the innate immune system (Sali et al., 2019). Monocytes and macrophages produce large amounts of proinflammatory mediators, such as TNF-α and IL-6, and then lead to endotoxemia. Efforts to address these issues are in desperate need. Lots of researches have reported that the AMPs can bind to the LPS of *E. coli* (Dixon et al., 2008; Na et al., 2017). A previous study has demonstrated that IDR-1018 can induce chemokine responses and suppress the LPS-induced TNF-α response (Michal et al., 2010). Nevertheless, to the best of our knowledge, neither the capacity of binding to LPS directly nor the effect of D amino acid substitution has been expounded before. In this study, we found that both IDR-1018 and D1018 could bind to LPS directly, then initially interrupt the interaction of LPS with TLRs, and subsequently inhibit the release of LPS-induced TNF-α and IL-6 in RAW264.7 cells (Figure 4), thereby effectively protect cells from acute inflammatory injury. Whereas, despite the potent antimicrobial activity, 1018M and D1018M not only failed to interact with LPS, on the contrary, they facilitated the generating of proinflammation cytokines, which suggests that the I4, V5, A6, V7 and I9 are critical amino acid residues for LPS binding while the binding sites may block the TLRs affinity positions.

In this study, based on IDR-1018 and its derived peptide 1018M, D amino acid peptides D1018 and D1018M were designed. All the peptides showed potent antimicrobial activity against *E. coli*, with D1018M having higher activity than others. D1018 had lower cytotoxic effect but higher protease stability than D1018M. Moreover, D1018 was found to be more potent than other peptides at destroying bacterial cell wall, penetrating membrane, and disrupting genomic DNA. Additionally, IDR-1018 and D1018 could bind to LPS and then inhibit LPS-induced proinflammation response efficiently. Our findings suggest that D1018 is a promising candidate for further study as a new antibacterial and anti-endotoxin agent.

## Data availability statement

The original contributions presented in the study are included in the article/supplementary material, further inquiries can be directed to the corresponding author.

## Author contributions

XW conceived and designed the experiments. RW, XD, QW, ZZ, and XW carried out all the experiments. RW and XW contributed in writing. XW and JW contributed in funding acquisition. All authors contributed to the article and approved the submitted version.

## Funding

This study was funded by the National Natural Science Foundation of China (No. 82002190).

## Conflict of interest

The authors declare that the research was conducted in the absence of any commercial or financial relationships that could be construed as a potential conflict of interest.

## Publisher’s note

All claims expressed in this article are solely those of the authors and do not necessarily represent those of their affiliated organizations, or those of the publisher, the editors and the reviewers. Any product that may be evaluated in this article, or claim that may be made by its manufacturer, is not guaranteed or endorsed by the publisher.
